# Carbon storage in US wetlands

**DOI:** 10.1038/ncomms13835

**Published:** 2016-12-13

**Authors:** A. M. Nahlik, M. S. Fennessy

**Affiliations:** 1 Department of Biology, Kenyon College, 202 N College Road, Gambier, Ohio 43022, USA

## Abstract

Wetland soils contain some of the highest stores of soil carbon in the biosphere. However, there is little understanding of the quantity and distribution of carbon stored in our remaining wetlands or of the potential effects of human disturbance on these stocks. Here we use field data from the 2011 National Wetland Condition Assessment to provide unbiased estimates of soil carbon stocks for wetlands at regional and national scales. We find that wetlands in the conterminous United States store a total of 11.52 PgC, much of which is within soils deeper than 30 cm. Freshwater inland wetlands, in part due to their substantial areal extent, hold nearly ten-fold more carbon than tidal saltwater sites—indicating their importance in regional carbon storage. Our data suggest a possible relationship between carbon stocks and anthropogenic disturbance. These data highlight the need to protect wetlands to mitigate the risk of avoidable contributions to climate change.

## Abstract (web summary)

Wetlands store large quantities of carbon, the distribution and quantity of which is little known. Here, Nahlik and Fennessy use data collected as part of the 2011 National Wetland Condition Assessment to estimate wetland carbon stocks across the United States, illustrating total storage of 11.52 PgC.

Soil carbon is vital in regulating climate, water supplies and biodiversity—all essential contributions to the provision of ecosystem services^1^. Wetlands contain a disproportionate amount of the earth's total soil carbon; holding between 20 and 30% of the estimated 1,500 Pg of global soil carbon^2^ despite occupying 5–8% of its land surface^3^. The anoxic conditions characteristic of wetland soils slow decomposition and lead to the accumulation of organic matter. As a result, wetlands can accumulate large carbon stores, making them an important sink for atmospheric carbon dioxide and holding up to or, in some cases, even more than 40% soil carbon^4^, which is substantially greater than the 0.5–2% carbon commonly found in agricultural soils^5^. In the United States, more than half of the historical wetland area has been lost due to anthropogenic activities^6^ resulting in a net transfer of carbon from the soil to the atmosphere^7^. This is particularly true for freshwater inland wetlands that make up most of the wetland area comprising, for example, 95% of all wetlands in the conterminous United States^8^,^9^. Many studies have focused on quantifying the carbon held in terrestrial ecosystems (so-called green carbon) and, more recently, on the carbon held in tidal saline ecosystems, often referred to as blue carbon^10^,^11^,^12^; however, our knowledge of carbon stored in inland freshwater wetlands, which we refer to here as teal carbon, is often overlooked or limited to site-specific studies. Accurate carbon accounting in wetlands is vital to reduce the risk of climate change contributions by identifying and protecting wetlands or wetland-dominated landscapes that hold disproportionately large carbon stocks, and to allow the inclusion of wetlands in carbon-offset programs, such as the United Nation's programme Reducing Emissions from Deforestation and Forest Degradation (UN-REDD+)^13^.

Here we provide a quantitative, robust estimate of wetland carbon storage in the conterminous United States as a function of soil depth, landscape position (inland versus tidal saline (that is, coastal)), and region, and an indication of how these stocks may be impacted by anthropogenic stressors using data from the US Environmental Protection Agency's (USEPA) 2011 National Wetland Condition Assessment (NWCA)^14^. These data provide empirical, unbiased, population-level estimates of soil carbon stocks with known confidence limits for targeted populations of wetlands at the national scale, and are not compiled based on the assumptions of a review of multiple sources, as earlier estimates have been (for example, ref. 8). We find that wetlands in the conterminous United States store a total of 11.52 PgC. Much of this carbon is stored within soil layers deeper than 30 cm and in freshwater inland wetlands—particularly those in the Midwest where wetlands with deep organic soils commonly occur in the northern tier states. Our data show that freshwater inland wetlands hold nearly 10-fold more carbon than the tidal saltwater sites that were assessed, in part due to the extensive area of inland wetlands compared with coastal sites—indicating their importance in regional carbon storage. Although we are unable to determine causality, our data also show that carbon stocks are significantly lower at wetland sites with most anthropogenic disturbance compared with sites with intermediate or least disturbance.

## Results

### National carbon stocks

**Figure 1:**
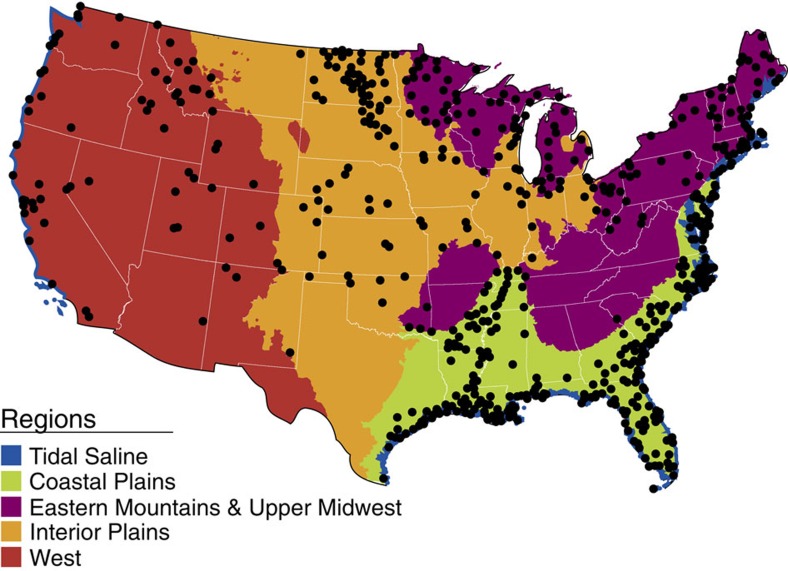
Map of the distribution of wetland probability sites. Sites (black points) were sampled as part of the US Environmental Protection Agency's 2011 National Wetland Condition Assessment (NWCA) and were analysed by five regions, Tidal Saline (blue area), Coastal Plains (green area), Eastern Mountains and Upper Midwest (purple area), Interior Plains (orange area) and West (red area).

To quantify carbon stocks (PgC), soil organic carbon concentration and bulk density data were collected by horizon from 120 cm-deep soil pits at 967 wetland sites across the conterminous United States (Fig. 1). Sites were selected from broadly defined NWCA Wetland Types (Table 1) using a stratified-random, probabilistic sampling design^15^,^16^ (the Generalized Random Tessellation Stratified survey^17^). These sites, known as the inference population, represent 25 million hectares of wetlands in the conterminous United States and store a total of 7.54±0.59 PgC (Table 2). The survey design, however, targeted a total of 38.4 million hectares, 13.4 million hectares of which (or 35%) could not be directly sampled primarily due to logistical difficulties^16^. Extrapolating to this full target population requires the assumption that the unsampled area follows the same trends as the sampled area. Accepting this assumption and scaling the estimate to the full 38.4 million hectares of this target population, we estimate that these wetlands store 11.52 PgC (Table 2), or close to 1% of the world's total soil organic carbon^2^.

### Geographic patterns in carbon stocks

**Figure 2:**
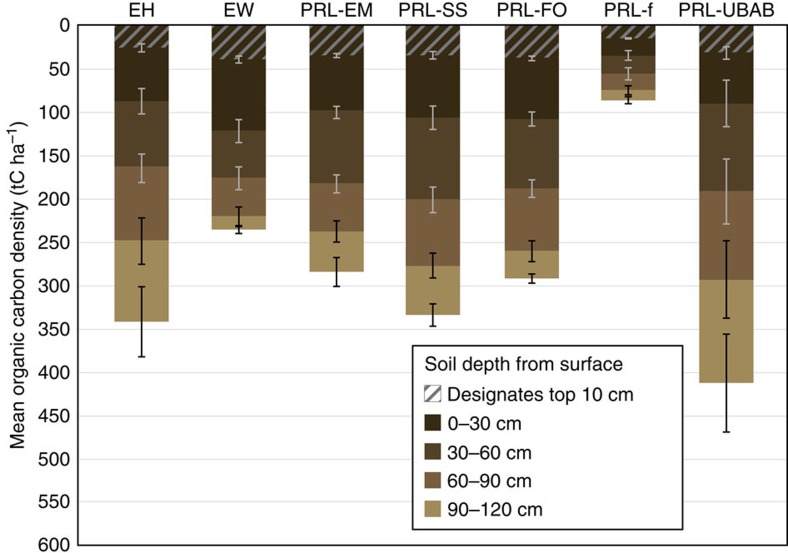
Mean soil organic carbon density to a depth of 120 cm by National Wetland Condition Assessment Wetland Type for wetlands of the conterminous United States. Carbon densities are reported as tC ha^−1^. National Wetland Condition Assessment (NWCA) Wetland Types include estuarine emergent (EH), estuarine woody (EW), palustrine, riverine and lacustrine emergent (PRL-EM), palustrine, riverine and lacustrine shrub (PRL-SS), palustrine, riverine and lacustrine forested (PRL-FO), palustrine, riverine and lacustrine farmed (PRL-f), palustrine, riverine and lacustrine unconsolidated bottom and aquatic bed (PRL-UBAB). The grey hatch within the bars represents the top 10 cm of the soil profile (within the 0–30 cm depth increment), followed by progressively lighter shading to represent 0–30, 30–60, 60–90 and 90–120 cm soil depths from the surface. Error bars (both white and black) represent s.e.m. Numerical values for this figure are presented in Supplementary Table 5.

**Figure 3:**
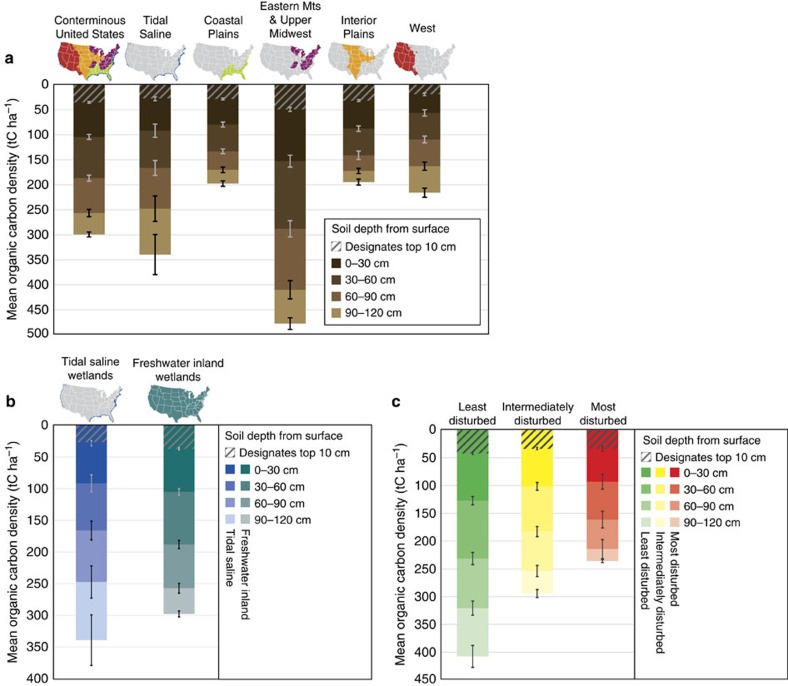
Mean soil organic carbon density to a depth of 120 cm for different subpopulations. Carbon densities (tC ha^−1^) are shown for (**a**) the nation and in five regions, (**b**) tidal saline wetlands (blue) and freshwater inland (teal) wetlands and (**c**) least (green), intermediately (yellow) and most disturbed (red) wetlands. Wetland geographic regions include Tidal Saline (TS; coastal and estuarine), Coastal Plains (CPL), Eastern Mountains and Upper Midwest (EMU), Interior Plains (IPL) and West (W). The grey hatch within the bars represents the top 10 cm of the soil profile (within the 0–30 cm depth increment), followed by progressively lighter shading to represent 0–30, 30–60, 60–90 and 90–120 cm soil depths from the surface. Note the data shown in **b**,**c** are calculated using the data shown in **a**. For 0–10, 0–30, 30–60, 60–90 and 90–120 cm, respectively, the number of samples (*n*) for each subpopulation (identified in subscript after the *n*) were as follows: *n*_national_=856, 853, 785, 590 and 435, *n*_ts_=282, 282, 270, 191 and 127, *n*_cpl_=212, 211, 181, 139 and 110, *n*_emu_=137, 135, 125, 99 and 71, *n*_ipl_=109, 109, 97, 71 and 57 and *n*_w_=116, 116, 112, 90 and 70. For tidal saline wetlands, *n*=282, 282, 270, 191 and 127 and for freshwater inland wetlands, *n*=574, 571, 515, 399 and 308, for 0–10, 0–30, 30–60, 60–90 and 90–120 cm, respectively. *n*_least disturbed_=173, 172, 164, 105 and 69, *n*_intermediately disturbed_=404, 404, 363, 278 and 193 and *n*_most disturbed_=279, 277, 258, 207 and 173 for 0–10, 0–30, 30–60, 60–90 and 90–120 cm, respectively. Error bars (both white and black) represent s.e.m. Numerical values for this figure are presented in Supplementary Table 5.

Carbon density (tC ha^−1^) and stocks varied as a function of location and wetland type (Fig. 2), which are intrinsically linked^18^. When grouped by region, carbon densities reflect a high degree of variability, ranging from 195 to 478 tC ha^−1^ (Fig. 3a). Wetlands of the Eastern Mountains and Upper Midwest store the most carbon, averaging 478±58 tC ha^−1^ and accounting for nearly half of the wetland carbon in the United States (Table 2). This is consistent with the abundance of wetlands with deep organic soils in the northern tier states where characteristic cool temperatures provide climatic conditions that can promote carbon accumulation. Of the 95 freshwater inland sites sampled with predominantly organic soils—designated as such if field descriptions of soil layers indicated that histosols were present^19^—half (47 sites) occurred in the Eastern Mountains and Upper Midwest region, storing an average of 539±47 tC ha^−1^ in the top 100 cm of soil—a conservative estimate given that many organic soil and peat deposits are >1 m deep^4^. The smallest wetland carbon pools were found in the Interior Plains (195±25 tC ha^−1^), where hydrologic modification and agricultural disturbance are extensive, contributing to wetland loss and degradation^20^ and effectively reducing soil organic carbon^21^. The Coastal Plains and West, where warm mean temperatures and low precipitation lead to more frequent dry downs^22^ and slower carbon sequestration rates, hold 198±21 and 216±30 tC ha^−1^, respectively. In all regions, the greatest carbon densities were found in the top 30 cm of the soil profile (Fig. 3a, Table 2). However, soil layers below 30 cm deep contain substantial cumulative reservoirs of carbon, with 65% of the total wetland soil carbon stored between 30 and 120 cm.

### Comparison of blue and teal carbon stocks

While recent work has focused on the power of salt marshes and mangroves (tidal saline wetlands) to accumulate blue carbon, less attention has been given to inland wetlands (teal carbon). Differences in carbon densities between saline and inland sites were surprisingly small, with the greatest difference between 91 and 120 cm, where tidal saline sites held more than twice as much carbon as freshwater sites on an areal basis (92±40 versus 41±5 tC ha^−1^; Fig. 3b). Carbon distribution was also more uniform with depth in the tidal saline sites, with about 25% of the total carbon pool in each of the four depth increments. Carbon densities in the inland sites decreased steadily with depth, from 35.3% of the total carbon in the top 30 cm to 13.6% between 91 and 120 cm. Although rates of carbon accretion cannot be inferred from these data, the smaller differences in the shallow soil layers compared with deeper layers in the tidal sites may be a result of insufficient time to compound the effects of annual differences in carbon accretion rates in the shallow soil layers. Unlike many inland wetlands, the on-going delivery of sediment and allochthonous carbon in tidal systems leads to sediment deposition, the burial of organic matter, and the vertical accretion of marsh surfaces, countering sediment compaction and subsidence that occurs deeper in the soil profile thus allowing carbon to accumulate over long time periods^12^. Increasing rates of sea level rise can also contribute to soil accretion in salt marshes by increasing the duration of tidal inundation and increasing sediment deposition on marsh surfaces^23^. Despite this, there is nearly 12 times the amount of estimated teal carbon as there is blue carbon in the conterminous United States due to the sheer area of inland wetlands (91% of total wetland area) compared with tidal sites (Table 2). It should be noted that our estimate does not account for the blue carbon held in subaqueous soil systems such as seagrass beds, which occur at water depths not sampled in this study; the inclusion of seagrass beds and their carbon stores would increase our estimate of blue carbon. Although estimates of the amount of carbon in US seagrass beds are lacking, the global average soil carbon stock reported for seagrasses (140 tC ha^−1^) is substantially lower than those for mangrove (471 tC ha^−1^) or salt marsh ecosystems (340 tC ha^−1^)^24^, which were included in our estimates and whose values are similar to what we report for tidal saline wetlands (340 tC ha^−1^). In this study, tidal sites overall account for 9% of the wetland area sampled and hold about 8% of the wetland carbon in the United States, illustrating the power of freshwater, inland wetlands to store carbon.

### Relationship between disturbance and carbon storage

To assess the impact of anthropogenic disturbance on soil carbon, the NWCA categorized sites as least, intermediately, or most disturbed using *a priori* defined indicators of physical, chemical and biological stressors that were observable at the time of the site visit, either in the wetland area assessed or the 100 m radius buffer area surrounding it (Table 3)^15^,^16^. The selected stressor indicators have a strong association with anthropogenic impacts and included several related to hydrologic alteration (such as the presence of ditches, dikes, or levees), or the occurrence of agricultural or urban land cover in the buffer area. Least disturbed sites, defined as those with the best available physical, chemical and biological condition given the current status of the landscape^25^, were those with few or no observed stressors. They had significantly higher soil carbon stocks (407±51 tC ha^−1^) than the most disturbed sites (236±47 tC ha^−1^; Fig. 3c). We lack information to determine whether humans have historically avoided developing the wettest sites with potentially higher overall carbon stores. If so, this pattern of human settlement might predispose least disturbed sites to have greater carbon densities. However, there is also historical evidence that even large deepwater wetlands with high carbon soils were effectively drained early in the history of US agricultural development, such as the Great Black Swamp in northwestern Ohio that covered 4,000 km^2^ with water levels up to 1-m deep (ref. 3). Despite this uncertainty in the pattern of anthropogenic disturbance, the mean difference of 171 tC ha^−1^ between least and most disturbed sites may represent a conservative estimate of carbon losses from human activities, as it is probable that even least disturbed sites have sustained some level of anthropogenic influence (for example, beyond the sampling site, such as in the greater wetland area or watershed) that could alter soil composition. For example, agricultural land use and the presence of tile drains in the drainage basins of the US Corn Belt region are shown to increase both stream and base flows, thereby increasing the annual discharge from that drainage basin^26^. This can lead to lower (that is, drier) regional groundwater levels that, over time, could increase soil carbon oxidation and affect soil carbon stores—even in wetland sites that lack directly observable stressors.

Although the mechanisms are not well understood, the deepest soil layers sampled (90–120 cm) had the greatest differences in soil carbon with 87±20, 40±7 and 22±3 tC ha^−1^ in least, intermediately and most disturbed wetlands, respectively (noting that the bulk density of 70% of the samples below 75 cm were estimated using a general boosted model with an *R*^2^ of 0.83 (see Methods)). The loss of carbon from deep in the soil profile may indicate that human impacts are not limited to surface and near-surface soil horizons, or it may be an artefact of the pattern of human settlement on the landscape, in which the wettest sites that tend to contain high levels of soil carbon were preferentially avoided. While anthropogenic disturbance has been reported to reduce carbon stocks to depths of a metre or more in tidal systems^27^, there are few corresponding data for freshwater wetlands. The pattern shown here indicating that human impacts may decrease carbon stocks across all wetland classes at the national scale will require further investigation.

## Discussion

Our study provides three important insights into wetland carbon dynamics and linkages to climate policy. First, our estimates of regional carbon stocks and carbon density are the only estimates based on unbiased, large-scale regional sampling that are extrapolated to a population of wetlands. Our data provide an important baseline for repeated future surveys, such as the 2016 NWCA, to track spatial and temporal trends in carbon stocks at the population scale. The data we provide here are also necessary to effectively identify characteristics of wetlands or types of wetlands in particular geographic areas that contain disproportionately large and regionally variable carbon stores if we are to implement policies related to climate protection. Interest in establishing markets for carbon credits based on wetland conservation and restoration activities is increasing in the US Federal Agencies, particularly for coastal wetlands^28^. For example, the state of California has initiated a carbon market that includes credits generated for carbon sequestration in wetlands^29^. Although we measured carbon stocks and not sequestration, large-scale wetland studies, such as the NWCA, could serve as an important basis for identifying areas with high-carbon wetlands for inclusion in climate policies. Our data indicate that freshwater inland sites, especially those with high carbon densities, which cumulatively store over 90% of the wetland soil carbon in the conterminous United States (10.67 of the estimated 11.52 PgC in the target population), could be viable candidates when establishing policy to preserve stored carbon that could otherwise, upon wetland drainage or degradation, enter the atmosphere. Wetland areas that seem particularly feasible targets for protecting carbon include the Coastal Plains, which has a regional store of 3.39 PgC, and the Eastern Mountains and Upper Midwest, where wetlands dominated by organic soils alone store 3.52 PgC. By comparison, mineral-soil wetlands for the same region store 1.21 PgC, and all tidal saline wetlands (mineral- and organic-soil combined) store 0.87 PgC (Table 2).

Secondly, we measure and account for deep carbon in this study. Limiting carbon stock estimates to the upper soil profile (for example, 0–30 cm) vastly underestimates wetland storage. Hansen and Nestlerode^30^ reflect this in their study where they report soil carbon densities to a depth of 10–15 cm in the Gulf of Mexico coastal region of 34–47 tC ha^−1^. Our measurements indicate that coastal carbon estimates may in fact be an order of magnitude greater, 340±94 tC ha^−1^, by assessing soils to 120 cm. Accounting for the carbon stocks of deeper soil layers more fully represents this ecosystem service that wetlands provide.

Finally, our results suggest that there may be a negative relationship between anthropogenic disturbance and soil carbon, perhaps extending to the deeper soil layers where we tend not to measure. One concern centred on wetlands, particularly freshwater sites, is that they are significant methane sources relative to coastal sites where high sulfate levels keep methane production low^31^. However, focusing on current rates of carbon fluxes overlooks the fact that wetland conversion, degradation and warming can lead to a rapid loss of ancient carbon^12^ that forms some of the large carbon pools documented in this study. For example, estimates show that the conversion of peatlands to other land uses could release the equivalent of 175–500 years of methane emissions if that same area were destroyed^32^. Sharp increases in carbon dioxide emissions have been noted in coastal wetlands with ecosystem degradation or conversion, amounting to 0.15–1.02 PgC globally^27^. The studies suggest a mechanistic explanation of how human activities could decrease soil carbon at regional scales, moving carbon from soil to the atmosphere as carbon dioxide and methane. Efforts to protect climate should address the role of wetlands as climate regulators and include measures for the conservation and sustainable management of their carbon stocks.

## Methods

### Sample frame

During the 2011 growing season (April–September, location dependent), 967 wetland points in the conterminous United States were sampled as part of the NWCA—an effort to evaluate the condition of the wetlands in the United States led by the USEPA with cooperation from state and tribal partners (Fig. 1). The target population was defined as: all wetlands of the conterminous United States not currently in crop production, including tidal and non-tidal wetted areas with rooted vegetation and, when present, shallow, open water <1 m in depth^15^. A probabilistic design was used to select wetland points using the US Fish & Wildlife Service's National Wetland Status & Trends (S&T) sample frame^2^,^9^,^33^, made up of ∼5,000 4-mi^2^ plots, and a Generalized Random Tessellation Stratified (GRTS) survey design^17^ stratified by state with unequal probability of selection by seven NWCA Wetland Types based on the S&T wetland categories (Table 1). Although S&T estimated wetland extent to be 44.6 million hectares (110.1 million acres) in the conterminous United States^9^,^33^, only a subset of wetlands included in S&T—approximately 38.4 million hectares (94.9 million acres)—met the NWCA target definition and so were included for sampling. The approximate 6.2 million hectares of wetlands included in S&T but were considered non-target for the NWCA, and therefore excluded from the survey, comprises wetlands that were actively cropped, wetlands used for aquaculture and wetlands that typically lack vegetation or routinely occur in water >1 m deep (for example, estuarine intertidal aquatic bed (E2AB), estuarine intertidal unconsolidated shore (E2US), marine intertidal (M2) and palustrine unconsolidated shore (PUS) S&T wetland categories (with S&T mapping codes followed in parentheses)). Of the 38.4 million hectares of NWCA target wetlands, a further 28% were unable to be sampled in the field due to landowner access denial, physical inaccessibility, size not meeting the minimum criteria, depth exceeding 1 m and so on. Therefore, the sampled wetland population for which we were able to directly extrapolate to (called the inference population) includes 25.1 million hectares (62.2 million acres; Supplementary Fig. 1).

### Field sampling

At each wetland point, a 0.5-ha circular assessment area (AA) was established, with no more than 10% of the area in upland or in water over 1 m deep. To meet the establishment criteria, the AA was occasionally adjusted to fit the shape of the wetland or reduced in size (to a minimum of 0.1 ha) if the point fell in a wetland smaller than 0.5 ha. In addition, a buffer area was established using 100-m transects at the cardinal directions of the AA perimeter. During a single-day visit to each wetland point, field crews collected data and samples associated with vegetation, soils, hydrology, water chemistry, algae and buffer according to the NWCA field protocol^15^.

Four 60 cm soil pits were excavated within the AA, after which a representative soil pit was established among the four and was expanded to 125 cm deep. At the representative soil pit, soil profiles were described by horizon to 125 cm or the deepest attainable depth. Specifically, soil textures were designated for each horizon, including information used to distinguish mineral soils (for example, sandy, loamy/clayey, mucky mineral) from organic soils (for example, peat, muck, mucky peat). For every horizon greater than 8 cm thick, a set of three hammered cores was collected for bulk density using a closed-top corer of a known volume (typically 6.5 cm in diameter and 4.5 cm in depth, although field crews could use improvised corers as long as the diameter and depth of the device was recorded), and an additional 1.0–2.5 l of soil for chemical analysis was collected. In saturated or inundated soils, special tools and alternate extraction methods were used to collect soil samples. Specifically, coffer dams reinforced with plastic and hand pumps were used to remove standing water from in and around soil pits, and King soil extractors (also known as tube extractors) were used to collect soil samples^15^. Upon collection, soil samples were refrigerated and sent in batches within 2 weeks to the Natural Resources Conservation Service (NRCS) laboratory in Lincoln, Nebraska for analysis. Standard NRCS Soil Survey Laboratory (SSL) procedures^34^,^35^ were used for analysis of sand, silt and clay, carbonate, total carbon, cation exchange capacity (CEC), electrical conductivity (EC) and bulk density (Supplementary Table 1). Soil organic carbon (SOC) was calculated as the difference between total and inorganic carbon. To prepare samples for carbon analysis, soils were air dried, crushed and sieved to <2 mm to obtain the fine earth fraction. Total carbon was measured using an elemental analyzer, and inorganic carbon (that is, calcium carbonate (CaCO_3_) equivalent) was determined by exposing the soils to hydrochloric acid (HCl) and measuring the evolved carbon dioxide (CO_2_) manometrically^34^.

### Quality assurance and bulk density modelling

Of the 4,061 soil horizons described, ∼25% were <8 cm thick and, therefore, were not sampled for analysis. Where soil carbon data from the top horizon were missing, it was equated to the next lower horizon (noting that if the top horizon was organic and the next lower horizon was mineral, the carbon content of the top horizon might be an underestimate, making this estimate conservative). Missing soil carbon from a middle horizon was estimated using the average of the horizon immediately above and below. Furthermore, ∼30% of the bulk density data were missing due to difficulties in the field or failed quality assurance. Bulk density for missing horizons and for measured values >2.0 g cm^−3^ (the latter assumed to be in error since 2.0 g cm^−3^ is the upper limit of measurable bulk density) was modelled using a generalized boosted model in the gbm R package^36^,^37^. Generalized Boosted Regression Modeling is a type of regression model that combines regression trees and boosting algorithms and is a means of predictive modelling by building many regression trees using an independently drawn, random sample, with each new tree using the prediction residuals from all preceding trees. Martin *et al*.^38^ showed that the Generalized Boosted Regression Model method produced more accurate and precise estimations of bulk density than a multiple regression, which is more commonly used. In building our model, we optimized the parameters using procedures described by Martin *et al*.^38^ and Jalabert *et al*.^39^. Seventy percent of the data were used to train the model. Model variables included (with percent of variability explained) SOC (77.2%), 10 NWCA Reporting Groups (4.6%, see the following section for more information on NWCA Reporting Groups), EC (3.7%), CEC (3.1%), horizon depth (2.8%), percent clay (2.2%), percent silt (2.1%), hydrogeomorphic (HGM) class (as determined in the field, 1.9%, ref. 15), percent sand (1.8%) and order of horizon within the profile (0.49%). The quality of the fit of the model (*R*^2^), tested against the remaining 30% of the data not used for model calibration, was 0.83 (Supplementary Fig. 2). Because of difficulties accurately sampling bulk density in the field, any measured values that differed from the modelled bulk density by 40% were replaced with the modelled value.

Sample sizes tended to decrease with horizon depth due to the difficulty extracting samples from deep horizons in the field. Of the 1,287 soil layers 75 cm deep or greater that were described by the field crews, 899 bulk density values were modelled. Most of these values necessitated modelling because the horizon was unable to be collected; only 13 bulk density values were modelled because the percent difference was greater than 40% between measured and modelled values, and 14 bulk density values were removed because they failed quality assurance.

Ultimately, 3,542 soil horizons had complete data on SOC and soil bulk density, which were used to calculate the concentration of stored carbon in each soil horizon using the following equation:


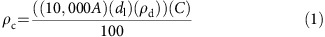


where, *ρ*_c_ is carbon density expressed in g m^−2^, *A* is area expressed in cm^2^ m^−2^, *d*_l_ is layer depth expressed in cm, *ρ*_d_ is bulk density expressed in g cm^−3^ and *C* is SOC concentration expressed as a percent.

Because the depths of soil horizons are not consistent among wetland soils, the quantity of stored carbon was calculated by dividing each horizon into 1 cm increments to allow us to report wetland carbon stocks within any depth range. We report depth up to 120 cm.

To summarize, sources of error in our analysis are predominantly associated with the fact that of the total 4,961 soil horizons described, ∼25% of these were <8 cm thick and, therefore, were not sampled for laboratory analysis. As a result of missing soil chemistry data for some layers, we extrapolated estimates of SOC to layers not measured from adjacent layers that had data. This tends to underestimate carbon content, particularly when the extrapolation was made for the top horizon using the underlying horizon. Second, missing bulk density values were estimated using generalized boosted regression modelling. While the fit of the model was strong (*R*^2^=0.83), this approach may introduce error, particularly for soil layers below 75 cm where a high proportion of bulk density values were modelled.

### Determination of organic and mineral soil carbon density

Carbon density in the top 100 cm of soil was estimated for organic- and mineral-soil dominated wetlands (that had soil carbon and bulk density values for every described layer up to 100 cm deep) located in inland (freshwater) and coastal (tidal saline) settings (Supplementary Table 2) using R statistical computing language^36^. Using the US soil taxonomy of Histosols^19^, organic-soil wetlands were designated as such if each horizon up to a minimum of 40 cm was identified in the field as an organic soil (for example, peat, muck or mucky peat), or at least 40 cm of the top 80 cm of soils were identified as organic, or, in the case of the presence of an impenetrable layer within the top 40 cm, two-thirds or more of the total soil thickness was identified as organic with <10 cm of total mineral soil. Mineral-soil wetlands were designated as such if they did not pass the criteria of an organic-soil wetland.

### Population estimates and reporting groups

The probabilistic design frame allows sample weights to be assigned to each individual site based on the inverse probability of that point being sampled^40^,^41^,^42^ so that results may be expressed as estimates of the entire resource by wetland area of sampled wetlands—25.2 million hectares (Supplementary Table 3, for example, ref. 16). The statistical estimates of mean and total carbon stocks for the national population of target wetlands were completed using the spsurvey R package^36^,^43^.

Ten NWCA Reporting Groups were developed based on a combination of (1) four major ecoregions (based on aggregations of Omernik Level III Ecoregions^44^), which include Coastal Plains (CPL), Eastern Mountains and Upper Midwest (EMU), Interior Plains (IPL) and West (W), and (2) wetland type, which includes estuarine (E) woody (W), estuarine (E) herbaceous (H), inland woody and inland herbaceous). Inland wetlands include palustrine, riverine and lacustrine (PRL) wetlands. Tidal saline wetlands (which include estuaries, high and low tidal marshes, and other coastal (tidal saline) wetlands) are combined for the entire contiguous United States (ALL), therefore, only 10 NWCA Reporting Groups exist^16^—ALL-EW, ALL-EH, EMU-PRLW, EMU-PRLH, CPL-PRLW, CPL-PRLH, IPL-PRLW, IPL-PRLH, W-PRLW and W-PRLH. In this study, the 10 NWCA Reporting Groups are most often combined by vegetation type resulting in five reporting groups (that is, the four ecoregions plus Tidal Saline). It should be noted that the ten NWCA Reporting Groups were defined for reporting purposes after site selection (that is, the survey design) so that each reporting group held a large enough sample size to make data analysis robust.

To address questions of how soil carbon varies regionally, estimates of carbon stocks were made for several subpopulations, including five geographic areas (Tidal Saline, Coastal Plains, Eastern Mountains & Upper Midwest, Interior Plains and West), carbon type (tidal saline blue carbon and freshwater inland teal carbon), and disturbance level (least, intermediate and most disturbed). Note that subpopulations represent the same set of data expressed in different ways.

### Disturbance gradient

Only data from the 967 randomly selected probability sites were used to report results in this study. However, an additional 171 non-probability sites (defined as such because they were not included in the S&T sample frame and instead hand-picked by states or tribes to be sampled) were measured in the field using the standard NWCA field and laboratory protocol at the same time as the probability sites. Field and laboratory data from all 1,138 wetland points, representing both probability and non-probability sites, were used to define a disturbance gradient.

The disturbance gradient was developed by screening sites using variables that have a strong association with anthropogenic impacts. Ultimately, nine disturbance indices and a plant disturbance metric were developed based on observations within the site (that is, the AA and buffer), hydrologic variables, soil trace metal data and the cover of alien plant species^16^ (Table 3). For each of these ten measures of disturbance, a disturbance threshold was set and every site was screened to test for exceedance.

Because the extent of human disturbance can vary greatly among regions and wetland types, thresholds were set independently for each of the ten NWCA Reporting Groups (Supplementary Table 4). Initially, if any threshold was exceeded at a site, it was not considered a least disturbed reference site; however, for some thresholds in some NWCA Reporting Groups, there were an insufficient number of sites that did not exceed the thresholds. Specifically, inland herbaceous wetlands located in the Interior Plains (IPL) and West (W) ecoregions had the most relaxed thresholds^16^. When thresholds were relaxed, least disturbed was defined as sites with no or minimally observed human disturbance (as opposed to zero observable human disturbance). Ultimately, the least disturbed reference sites were those that were below the thresholds for all 10 measures.

Sites classified as most disturbed on the disturbance gradient were defined using a filtering process in the same manner as the least disturbed sites. In this case, thresholds were set for each measure to define high levels of disturbance. If any single threshold for any measure was exceeded, the site was considered a most disturbed site. Because most disturbed is a relative definition, ∼20–30% of the sites were defined as most disturbed, and thresholds were set accordingly.

Finally, the sites not falling into either least or most disturbed were classified into the intermediate disturbance category. Of 1,138 sites screened, 277 sites (24%) were classified as least disturbed, 530 sites (47%) were intermediately disturbed and 331 sites (29%) were most disturbed.

Note that of the 195 organic-soil wetlands in inland and coastal settings, 62 sites were defined as least disturbed (that is, 22% of all least disturbed sites were dominated by organic soil), 80 sites were intermediately disturbed (that is, 15% of all intermediately disturbed sites were dominated by organic soil) and 53 sites were most disturbed (that is, 16% of all most disturbed sites were dominated by organic soil), suggesting that anthropogenic disturbance may similarly affects carbon-rich, organic-soil wetlands and lower carbon, mineral-soil wetlands.

### Data availability

Data (raw data and general results from the 2011 National Wetland Condition Assessment) are publically available from https://www.epa.gov/national-aquatic-resource-surveys/data-national-aquatic-resource-surveys

## Additional information

**How to cite this article:** Nahlik, A. M. & Fennessy, M. S. Carbon storage in US wetlands. *Nat. Commun.* **7,** 13835 doi: 10.1038/ncomms13835 (2016).

**Publisher's note:** Springer Nature remains neutral with regard to jurisdictional claims in published maps and institutional affiliations.

## Supplementary Material

Supplementary Information Supplementary Figures, Supplementary Tables and Supplementary References.

## Figures and Tables

**Table 1:** Wetland types and descriptions sampled as part of the 2011 National Wetland Condition Assessment.

**NWCA wetland types**	**Based on**	
	**S&T categories**	**Description of wetlands included in NWCA**
EH—estuarine emergent	E2EM—estuarine intertidal emergent	Estuarine (E) intertidal emergent (that is, herbaceous=H) wetlands
EW—estuarine woody	E2SS—estuarine intertidal forest/shrub	Estuarine (E) intertidal forested and shrub (that is, woody=W) wetlands
PRL-EM—palustrine, riverine and lacustrine emergent	PEM—palustrine emergent	Emergent (EM) wetlands in palustrine, shallow riverine or shallow lacustrine littoral (PRL) settings
PRL-SS—palustrine, riverine and lacustrine shrub	PSS—palustrine shrub	Shrub-dominated (SS) wetlands in palustrine, shallow riverine or shallow lacustrine littoral (PRL) settings
PRL-FO—palustrine, riverine and lacustrine forested	PFO—palustrine forested	Forested (FO) wetlands in palustrine, shallow riverine or shallow lacustrine littoral (PRL) settings
PRL-f—palustrine, riverine and lacustrine farmed	Pf—palustrine farmed	Farmed (f) wetlands in palustrine, shallow riverine or shallow lacustrine littoral (PRL) settings; only includes a subset that is not currently in crop production
PRL-UBAB—palustrine, riverine and lacustrine unconsolidated bottom and aquatic bed	PUBPAB—palustrine unconsolidated bottom/aquatic bed	Open-water ponds and aquatic bed (UBAB) wetlands in palustrine, shallow riverine or shallow lacustrine littoral (PRL) settings

National Wetland Condition Assessment (NWCA) Wetland Types are cross-referenced with US Fish and Wildlife Service Status and Trends (S&T) Categories^9^,^16^ on which they are based.

**Table 2:** Estimated carbon stocks to a depth of 120 cm.

					**Sum**	**Area**
	**0–30 cm**	**31–60 cm**	**61–90 cm**	**91–120 cm**	**0–120 cm**	**(10**^**6**^** ha)**
*PgC stored by depth increment for the inference population*						
Conterminous United States	2.63±0.12	2.08±0.15	1.76±0.19	1.08±0.12	7.54±0.59	25.2
Region						
Tidal Saline	0.20±0.03	0.17±0.03	0.18±0.06	0.20±0.09	0.76±0.21	2.2
Coastal Plains	0.83±0.05	0.56±0.05	0.38±0.06	0.28±0.06	2.05±0.21	10.4
E. Mts & Upper Midw	1.24±0.10	1.09±0.13	0.98±0.15	0.55±0.09	3.86±0.47	8.1
Interior Plains	0.27±0.02	0.17±0.03	0.10±0.02	0.07±0.02	0.60±0.08	3.1
West	0.08±0.01	0.07 ±0.01	0.08±0.01	0.07±0.01	0.30±0.04	1.4
Carbon type						
Blue (tidal saline)	0.20±0.03	0.17±0.03	0.18±0.06	0.20±0.09	0.76±0.21	2.2
Teal (all others)	2.42±0.12	1.91±0.15	1.59±0.18	0.93±0.11	6.85±0.55	23.0
Disturbance category						
Least disturbed	0.70±0.04	0.58±0.06	0.49±0.07	0.48±0.11	2.25±0.28	5.5
Intermediate disturbed	1.29±0.09	1.04±0.11	0.90±0.13	0.52±0.09	3.75±0.42	12.7
Most disturbed	0.64±0.09	0.47±0.10	0.37±0.12	0.15±0.02	1.63±0.33	7.0
						
*PgC stored by depth increment for the target population*						
Conterminous United States	4.02	3.17	2.68	1.64	11.52	38.4
Region						
Tidal Saline	0.20	0.20	0.22	0.25	0.87	2.7
Coastal Plains	1.37	0.92	0.64	0.47	3.39	17.1
E. Mts & Upper Midw	1.53	1.35	1.22	0.68	4.78	10.0
Interior Plains	0.27	0.26	0.16	0.11	0.80	5.0
West	0.08	0.19	0.19	0.19	0.66	3.6
Carbon type						
Blue (tidal saline)	0.20	0.20	0.22	0.25	0.87	2.7
Teal (all others)	3.25	2.72	2.21	1.45	9.63	35.7

E. Mts & Upper Midw, Eastern Mountains and Upper Midwest.

Carbon stock estimates (PgC) for geographic regions, carbon type and disturbance category are provided for (a) the inference population and (b) the target population. Wetland area represented by each group is provided in 10^6^ ha. Means are presented with s.e.m. for the inference population. Means for disturbance category s.e.m. for all values are not presented for the target population data because they cannot be calculated for the wetland area not able to be sampled.

**Table 3:** Measures of disturbance used to define the disturbance gradient.

**Measure of disturbance**	**Data type**	**Index description**
Agriculture disturbances	Buffer	Number of proximity-weighted* observed agriculture disturbances within the buffer, including pasture/hay, row crops, irrigation, confined animal feeding operations and so on
Residential and urban disturbances	Buffer	Number of proximity-weighted observed residential and urban disturbances within the buffer, including roads, parking lots, golf courses, housing, trash, landfill, dumping and so on
Hydrologic disturbances	Buffer	Number of proximity-weighted observed hydrologic disturbances within the buffer, including ditching, dikes and dams, water level control structures, excavation, fill, riprap and so on
Industrial disturbances	Buffer	Number of proximity-weighted observed industrial disturbances within the buffer, including oil drilling, gas wells, mines (surface or underground) and military operations
Habitat modifications	Buffer	Number of proximity-weighted observed habitat modifications within the buffer, including clear cuts, tree plantations, mowing, highly grazed grasses, soil compaction, recent burning and so on
Buffer summary	Buffer	The summary of threshold scores from the buffer indices (agriculture, residential/urban, hydrologic, industrial, habitat)
High impact hydrologic disturbances	Hydrology	Number of observed high impact hydrologic disturbances within the AA, including damming features, impervious surfaces, pumps, pipes, culverts and so on
Moderate impact hydrologic disturbances	Hydrology	Number of observed moderate impact hydrologic disturbances within the AA, including shallow channels, animal trampling, vehicle ruts and so on
Soil heavy metal index	Soil Metal Content	Sum of the number of heavy metal concentrations (Ag, Cd, Co, Cr, Cu, Ni, Pb, Sb, Sn, V, W, Zn) measured in the uppermost horizon above published thresholds
Relative cover of alien plant species	Vegetation	Calculated percentage of relative cover of alien plant species† in the AA, measured within five 100 m^2^ plots

Modified from US Environmental Protection Agency's, 2011 National Wetland Condition Assessment Technical Report^16^.

^*^Buffer observations were recorded by proximity to the AA, with observed stressors closest to the AA receiving higher stressor scores than those farthest from the AA.

^†^Alien plant species are defined as those that are either introduced to the conterminous United States or are adventive to the location of occurrence.
