# Supplementary material for: Carbon storage in US wetlands
Source: Nat Commun. 2016 Dec 13;7:13835. doi: 10.1038/ncomms13835 (PMC5159918; doi:10.1038/ncomms13835)
Supplement: Supplementary Information — Supplementary Figures, Supplementary Tables and Supplementary References. [file ncomms13835-s1.pdf]

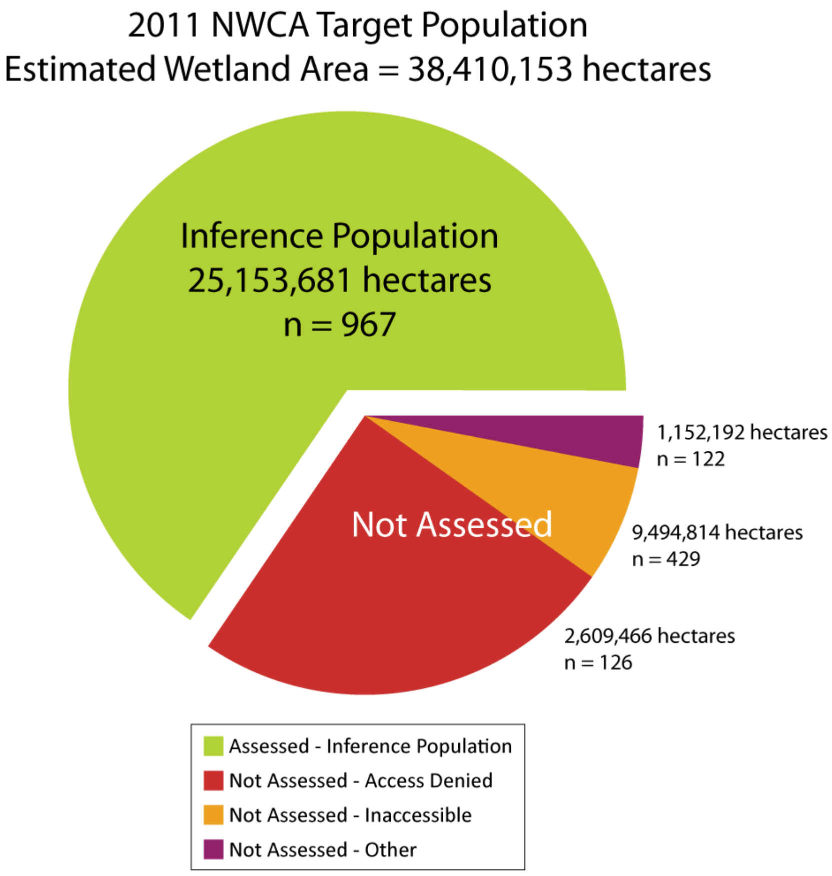


**Supplementary Figure 1 | Wetland area included in the US target wetland population sampled in the 2011 National Wetland Condition Assessment.** Of this, sites representing 28% of the area were unable to be sampled due to denied access (red), inaccessibility (orange), or other reasons (purple). The sampled wetland population (green) for which we were able to directly extrapolate estimates of carbon stocks is the 'inference population'. Modified from U.S. Environmental Protection Agency, *2011 National Wetland Condition Assessment Technical Report*[1].

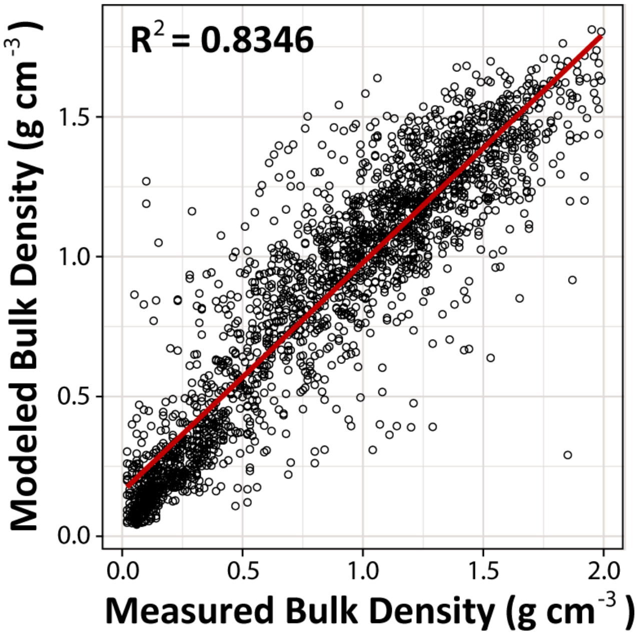


**Supplementary Figure 2 | Fit of the generalized boosted model used to predict bulk density.** Regression (red line) shows the measured bulk density (g cm$^{-3}$) compared to the modeled bulk density (g cm$^{-3}$).

**Supplementary Table 1 | Natural Resource Conservation Service Standard Soil Survey Laboratory Procedures.** Modified from U.S. Environmental Protection Agency, *2011 National Wetland Condition Assessment Laboratory Operations Manual*[2]. The acronyms used in the table include the following: MDL for method detection limit, PQL for practical quantitation limit, nd for not detectable, and n/a for not available.

| Method | Standard SSL Procedure | Analysis Description | Analyte | Units | MDL | PQL | Potential Sample Range | Accuracy Objective |
|---|---|---|---|---|---|---|---|---|
| **Particle Size Distribution Analysis, <2mm, air dry** | 3A1a1a | Gravimetric pipet analysis | Clay | % | na | na | 0 to 93.1 | n/a |
| | | | Silt | % | na | na | 0.1 to 100 | n/a |
| | | | Sand | % | na | na | 0 to 94.5 | n/a |
| **Calcium carbonate equivalent, <2mm** | 4E1a1a1a1 | Closed-system measurement of evolved $CO_2$ after acid treatment | $CaCO_3$ | % | 0.5 | 2.5 | nd to 105 | n/a |
| **Total Carbon** | 4H2a1-3 | Elemental analyzer | C | % | 0.04 | 0.2 | nd to 62.43 | 0.01% |
| **Cation exchange capacity (CEC)** | 4B1a1b1-4 | Displacement of cations after washing; distillation and titration | CEC | cmol(+) $kg^{-1}$ | 0.1 | 0.6 | nd to 252 | 0.1 cmol(+) kg-1 |
| **Electrical Conductivity (EC)** | 4F1a1a1a1 | Measurement using an electric bridge | EC | mmhos $cm^{-1}$ | 0.001 | 0.005 | nd to 167.4 | 0.01 mmhos cm-1 |
| **Bulk Density (Db)** | 3B1a-d | Volumetric oven-dry weight | Db | g $cm^{-3}$ | na | na | 0.06 to 2.53 | 0.01 $g/cm^{-3}$ |

**Supplementary Table 2 | Mean carbon densities for mineral-soil and organic-soil dominated wetlands in freshwater inland and coastal tidal saline locations.** The carbon densities (tC $ha^{-1}$) have been extrapolated to the inference population, with the area represented ($10^6$ ha) and the carbon stocks for the top 100 cm are reported (PgC). Note that the n-values reflect the subset of sites that had soil carbon and bulk density values for every described layer up to 100 cm deep. Means are presented with s.e.m.

| Description | Mean C Density for 0-100 cm depth (tC $ha^{-1}$) | n | Area Represented ($10^6$ ha) | PgC Stored in 0-100 cm depth |
|---|---|---|---|---|
| Tidal Saline, Mineral-Soil Wetlands | 172 ± 15 | 124 | 1.0 | 0.17 ± 0.01 |
| Freshwater Inland, Mineral-Soil Wetlands | 155 ± 9 | 317 | 15.8 | 2.45 ± 0.14 |
| Tidal Saline, Organic-Soil Wetlands | 619 ± 222 | 53 | 0.5 | 0.29 ± 0.10 |
| Freshwater Inland, Organic-Soil Wetlands | 615 ± 63 | 65 | 5.9 | 3.64 ± 0.37 |

**Supplementary Table 3 | Distribution of probability sites and acres represented in the 2011 National Wetland Condition Assessment.**

| NWCA Region | Number of Probability Sites Sampled | Hectares Represented by Sampled Probability Sites | Proportion of the Inference Population |
|---|---|---|---|
| **National** 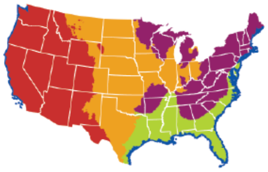 | 967 | 25,153,721 | 100% |
| **Tidal Saline** 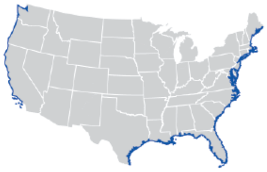 | 327 | 2,219,962 | 9% |
| **Coastal Plains** 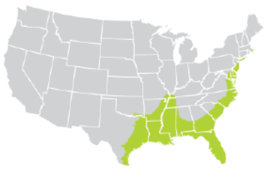 | 225 | 10,363,924 | 41% |
| **Eastern Mountains and Upper Midwest** 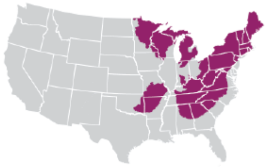 | 138 | 8,064,371 | 32% |
| **Interior Plains** 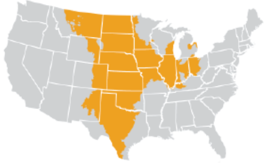 | 156 | 3,099,555 | 12% |
| **West** 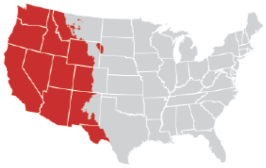 | 121 | 1,405,909 | 6% |

**Supplementary Table 4 | Least disturbed thresholds and most disturbed thresholds used to define sites along the disturbance gradient.** Least disturbed thresholds are denoted by green text (or the upper number in each cell) and most disturbed thresholds are denoted by red text (or the lower number in each cell). Thresholds were set by for individual NWCA Reporting Groups (location/wetland type/vegetation combinations), which include estuarine wooded for the contiguous US (ALL-EW), estuarine herbaceous vegetation for the contiguous US (ALL-EH), Coastal Plains palustrine, riverine, and lacustrine woody (CPL-PRLW), Coastal Plains palustrine, riverine, and lacustrine herbaceous (CPL-PRLH), Eastern Mountains and Upper Midwest palustrine, riverine, and lacustrine woody (EMU-PRLW), Eastern Mountains and Upper Midwest palustrine, riverine, and lacustrine herbaceous (EMU-PRLH), Interior Plains palustrine, riverine, and lacustrine woody (IPL-PRLW), Interior Plains palustrine, riverine, and lacustrine herbaceous (IPL-PRLH), West palustrine, riverine, and lacustrine woody (W-PRLW), and West palustrine, riverine, and lacustrine herbaceous (W-PRLH). See Fig. 1 of the main text for a map of regions. Threshold units are based on the number of observations of the measure of disturbance, described in the Index Description column in Table 3 of the main text, with the exception of Relative Cover of Alien Plant Species (percentages).

| Measure of Disturbance | ALL-EW | ALL-EH | CPL-PRLW | CPL-PRLH | EMU-PRLW | EMU-PRLH | IPL-PRLW | IPL-PRLH | W-PRLW | W-PRLH |
|---|---|---|---|---|---|---|---|---|---|---|
| **Agriculture Disturbances** | ≤0.00<br>≥0.25 | ≤0.00<br>≥0.25 | ≤0.00<br>≥0.25 | ≤0.00<br>≥0.60 | ≤0.00<br>≥0.25 | ≤0.00<br>≥0.30 | ≤0.10<br>≥0.30 | ≤0.15<br>≥0.60 | ≤0.10<br>≥0.60 | ≤0.60<br>≥0.75 |
| **Residential and Urban Disturbances** | ≤0.00<br>≥0.25 | ≤0.00<br>≥0.25 | ≤0.00<br>≥0.25 | ≤0.00<br>≥0.60 | ≤0.00<br>≥0.25 | ≤0.10<br>≥0.30 | ≤0.10<br>≥0.30 | ≤0.15<br>≥0.60 | ≤0.10<br>≥0.60 | ≤0.60<br>≥0.75 |
| **Hydrologic Disturbances** | ≤0.00<br>≥0.25 | ≤0.00<br>≥0.25 | ≤0.00<br>≥0.25 | ≤0.00<br>≥0.60 | ≤0.00<br>≥0.25 | ≤0.00<br>≥0.30 | ≤0.10<br>≥0.30 | ≤0.15<br>≥0.60 | ≤0.10<br>≥0.60 | ≤0.60<br>≥0.75 |
| **Industrial Disturbances** | ≤0.00<br>≥0.25 | ≤0.00<br>≥0.25 | ≤0.00<br>≥0.25 | ≤0.00<br>≥0.60 | ≤0.00<br>≥0.25 | ≤0.00<br>≥0.30 | ≤0.00<br>≥0.30 | ≤0.05<br>≥0.60 | ≤0.00<br>≥0.60 | ≤0.00<br>≥0.75 |
| **Habitat Modifications** | ≤0.00<br>≥0.25 | ≤0.00<br>≥0.25 | ≤0.00<br>≥0.50 | ≤0.20<br>≥1.00 | ≤0.00<br>≥0.50 | ≤0.10<br>≥0.60 | ≤0.20<br>≥0.60 | ≤0.15<br>≥1.20 | ≤0.10<br>≥0.80 | ≤1.00<br>≥1.50 |
| **Buffer Summary** | ≤0.00<br>≥0.75 | ≤0.00<br>≥0.75 | ≤0.00<br>≥1.00 | ≤0.20<br>≥1.50 | ≤0.00<br>≥1.00 | ≤0.10<br>≥1.00 | ≤0.20<br>≥1.00 | ≤0.30<br>≥1.80 | ≤0.10<br>≥1.00 | ≤1.20<br>≥2.50 |
| **High Impact Hydrologic Disturbances** | ≤0<br>≥1 | ≤0<br>≥1 | ≤0<br>≥1 | ≤0<br>≥2 | ≤0<br>≥1 | ≤0<br>≥2 | ≤0<br>≥1 | ≤1<br>≥1 | ≤0<br>≥2 | ≤1<br>≥3 |
| **Moderate Impact Hydrologic Disturbances** | ≤0<br>≥1 | ≤0<br>≥1 | ≤0<br>≥1 | ≤1<br>≥2 | ≤0<br>≥1 | ≤0<br>≥2 | ≤1<br>≥2 | ≤1<br>≥2 | ≤1<br>≥2 | ≤1<br>≥3 |
| **Soil Heavy Metal Index** | ≤0<br>≥2 | ≤0<br>≥2 | ≤0<br>≥2 | ≤0<br>≥2 | ≤0<br>≥2 | ≤1<br>≥2 | ≤2<br>≥2 | ≤2<br>≥2 | ≤2<br>≥3 | ≤1<br>≥3 |
| **Relative Cover of Alien Plant Species** | ≤5%<br>≥50% | ≤5%<br>≥50% | ≤5%<br>≥50% | ≤5%<br>≥50% | ≤5%<br>≥50% | ≤5%<br>≥50% | ≤5%<br>≥50% | ≤20%<br>≥50% | ≤5%<br>≥50% | ≤20%<br>≥50% |

**Supplementary Table 5 | Mean carbon densities by soil depth increments.** Carbon densities are reported as tC $ha^{-1}$. Means are presented with s.e.m. See Table 1 in the main text for definitions of wetland types. Graphical representations of this table are presented in Figs. 2 and 3 in the main text.

| | *0 – 10 cm* | *0 – 30 cm* | *31 – 60 cm* | *61 – 90 cm* | *91 – 120 cm* |
|---|---|---|---|---|---|
| *Conterminous US* | 35.6 ± 1.6 | 104.7 ± 4.9 | 82.5 ± 6.1 | 69.8 ± 7.4 | 42.8 ± 4.9 |
| **Region** | | | | | |
| *Tidal Saline* | 27.5 ± 4.2 | 92.3 ± 13.3 | 74.5 ± 14.8 | 81.4 ± 25.4 | 91.9 ± 40.0 |
| *Coastal Plains* | 29.0 ± 1.7 | 80.0 ± 5.0 | 53.6 ± 4.9 | 37.1 ± 5.5 | 27.3 ± 5.4 |
| *Eastern Mts & Upper Midw* | 49.5 ± 3.8 | 153.2 ± 12.0 | 135.3 ± 16.2 | 122.0 ± 18.3 | 67.6 ± 11.7 |
| *Interior Plains* | 32.3 ± 1.8 | 88.2 ± 5.5 | 53.3 ± 8.5 | 31.3 ± 5.3 | 22.3 ± 6.0 |
| *West* | 19.2 ± 2.2 | 56.7 ± 6.3 | 53.2 ± 6.8 | 53.4 ± 8.2 | 53.0 ± 9.1 |
| **Carbon Type** | | | | | |
| *Blue* | 27.5 ± 4.2 | 92.3 ± 13.3 | 74.5 ± 14.8 | 81.4 ± 25.4 | 91.9 ± 40.0 |
| *Teal* | 36.2 ± 1.7 | 105.6 ± 5.1 | 83.1 ± 6.5 | 69.2 ± 7.7 | 40.6 ± 4.6 |
| **Disturbance Category** | | | | | |
| *Least Disturbed-US* | 42.3 ± 2.2 | 127.2 ± 7.3 | 104.1 ± 10.9 | 89.3 ± 12.7 | 86.9 ± 19.6 |
| *Intermediate Disturbed-US* | 33.9 ± 2.3 | 101.4 ± 7.0 | 81.4 ± 8.9 | 70.9 ± 10.1 | 40.5 ± 7.2 |
| *Most Disturbed-US* | 33.9 ± 4.1 | 93.2 ± 13.2 | 68.0 ± 14.9 | 53.0 ± 17.0 | 21.6 ± 2.6 |
| **Wetland Type** | | | | | |
| *EH* | 25.9 ± 4.6 | 87.9 ± 14.7 | 77.5 ± 16.4 | 84.1 ± 26.8 | 93.2 ± 40.4 |
| *EW* | 38.5 ± 4.0 | 123.0 ± 13.4 | 54.5 ± 13.2 | 44.6 ± 11.3 | 14.7 ± 4.5 |
| *PRL-EM* | 34.7 ± 2.2 | 101.4 ± 7.1 | 82.6 ± 10.4 | 54.9 ± 12.3 | 46.8 ± 16.6 |
| *PRL-SS* | 34.4 ± 4.3 | 106.4 ± 13.5 | 94.8 ± 14.8 | 75.9 ± 14.3 | 57.1 ± 12.8 |
| *PRL-FO* | 37.9 ± 2.6 | 109.0 ± 8.0 | 80.4 ± 10.1 | 72.4 ± 12.0 | 31.6 ± 5.3 |
| *PRL-f* | 14.9 ± 0.6 | 35.1 ± 5.6 | 21.3 ± 7.0 | 18.9 ± 5.2 | 11.4 ± 4.1 |
| *PRL-UBAB* | 30.9 ± 7.1 | 89.7 ± 26.9 | 101.8 ± 37.5 | 101.6 ± 44.7 | 119.7 ± 56.5 |

## Supplementary References


1. U.S. Environmental Protection Agency, *2011 National Wetland Condition Assessment Technical Report* (EPA Publication 843-R-15-006, 2016), https://www.epa.gov/national-aquatic-resources-surveys/nwca
2. U.S. Environmental Protection Agency, *National Wetland Condition Assessment: Laboratory Operations Manual* (EPA Publication 843-R-10-002, 2016), https://www.epa.gov/national-aquatic-resources-surveys/nwca
